# American Journal of Ophthalmology

**Published:** 1862-11

**Authors:** 


AMERICAN JOURNAL OF OPHTHALMOLOGY.

THIS is a Bi-monthly, Edited by Julius Homberger, M. D,, No. 24 W. 12th street, N. Y., the second number of which is before us. It is a valuable Journal to all who take an interest in the department to which it pertains; and particularly to those engaged in this specialty of medical practice. Its matter is the emanation of those who have given their undivided attention to the structure of the eye and the treatment of its diseases. It is a work that all general physicians should have at hand and frequently consult, to the oculist it is of course a *sine qua non.*

We welcome the Journal of Ophthalmology to our exchange list, and shall always consult its pages with much interest.

T.

☞ "Having directed the attention to *some* of the defects in the use of language, it becomes proper and necessary, in concluding the subject, to suggest the remedy by which such defects alone can be overcome.

"The first step toward such a desirable consummation *is to cultivate a close and intimate acquaintance with the best writers of every age and every tongue.*"—Cosmos.

Moses and the Prophets! but that's a long STEP—a great deal longer than the two steps the little word "alone," up there, would have to take, to get back to where it belongs in the sentence.

